# Navigating the Landscape of Herniated Discs: A Rare Case of Herniated Disc Regression

**DOI:** 10.7759/cureus.51568

**Published:** 2024-01-03

**Authors:** Ilko Ilyov, Edvin Vasvi, Petar-Preslav Petrov, Vladislav Velchev, Plamen Penchev

**Affiliations:** 1 Department of Medicine, Medical University of Plovdiv, Plovdiv, BGR; 2 Department of Neurosurgery, St. Anna Hospital, Varna, BGR; 3 Department of Anatomy, Histology, and Embryology, Medical University of Plovdiv, Plovdiv, BGR

**Keywords:** progressive myelopathy, compressive myelopathy, mri, herniated disc, clinical case report

## Abstract

A herniated disc is a condition in which the nucleus pulposus is displaced from the intervertebral space. It usually leads to back pain, thus being the most common reason for it. Patients often describe the first symptoms of a herniated disc as extreme and decisive pain. Unlike the usual mechanical back pain, a herniated disc is often related to a stinging or burning sensation that often spreads to the lower extremities and proves to be continuous at lower temperatures. We present a case of a 58-year-old male patient who visited the Acibadem City Clinic with complaints of pain initially starting from his hip, which in time extended to his left leg (L5 radiculopathy) and a few days later to his right leg (L5 radiculopathy). Before visiting the clinic, he had been treated in Germany with physiotherapy and supplements, which had proved ineffective. After an MRI, which revealed an L4-L5 herniated disc, he underwent conservative treatment with nonsteroidal anti-inflammatory drugs (NSAIDs) and proton pump inhibitors (PPIs) for 14 days in addition to Medrol 4mg tablets (3x1 per day for 10 days). On the third day of the treatment, 60% of the symptoms had subsided. Seven months later, he came in for a scheduled checkup, and 95% of the symptoms were gone. A controlled MRI was done, and the herniated disc had completely vanished. We hope that this type of research will benefit medical professionals, patients, researchers, doctors, and students, among others. Such cases also contribute to the quality of care for such patients and help set regulated factual guidelines regarding their treatment as a whole.

## Introduction

Lumbar disc herniations are the most common cause of lower back pain, neurological dysfunction, and leg pain [1]. The pain associated with lumbar disc herniations is the result of the compression of the disc fragments onto the neural radix and can be accompanied by weakness of the muscles [1]. In 90%-95% of cases, they are in the region of L4-L5, L5-S1 [2]. It is important to keep in mind that most disc herniations do not have many symptoms and are usually found during an MRI [1-5]. Disc herniation is typically linked to disc degeneration. The disc fibrochondrocytes experience senescence and a decrease in proteoglycan synthesis as they age. The dehydration and disc collapse brought on by this reduction in proteoglycans increases the strain on the annulus fibrosus, causing tears and fissures, which in turn facilitate the nucleus pulposus herniation. As such, the gradual onset of chronic symptoms is caused by repetitive mechanical stressors applied to the disc [5].

This case report is consistent with the main takeaway, which emphasizes the value of informed decision-making and patient-centered care. The current trend in healthcare, which encourages shared decision-making between practitioners and patients, is in line with this emphasis on informed decision-making and offers insightful clinical information about how lumbar disc herniations present and are conservatively managed [3,4,5]. Clinicians handling cases similar to our patient can benefit from this thorough account of the patient's symptoms, course, and reaction to conservative treatment. Our purpose is to report the outcomes of conservative treatment and the full recovery of a lumbar disc herniation.

ChatGPT was used for additional assistance and help while writing the article, and Grammarly was used to correct grammar and punctuation errors.

## Case presentation

**Figure 1:**
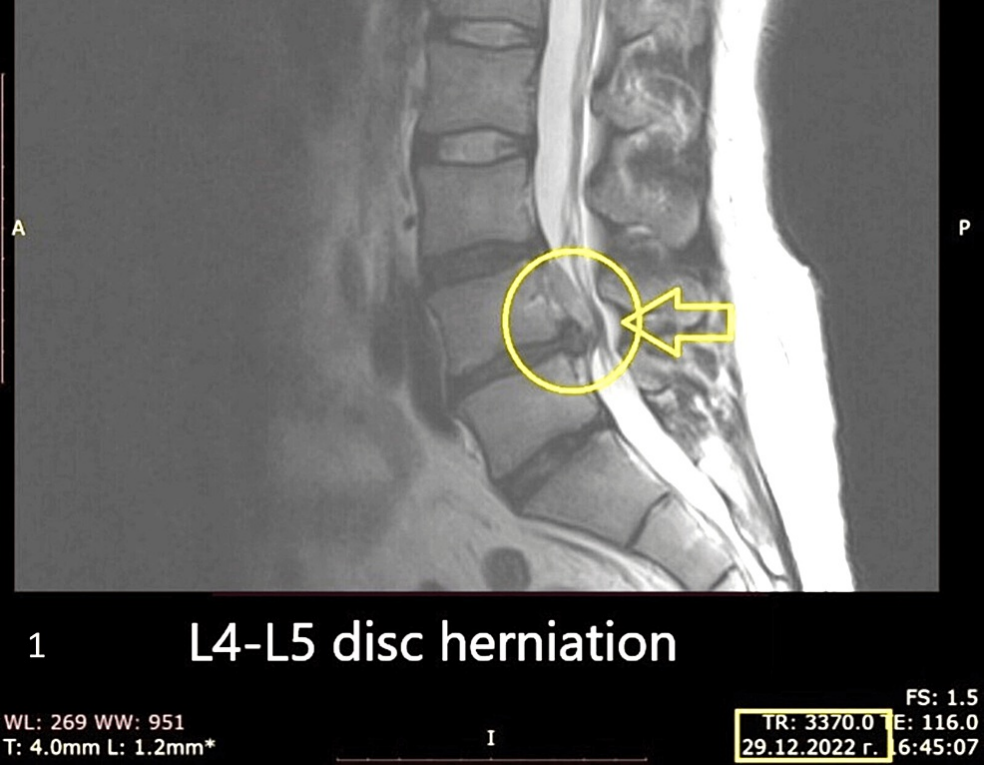
An MRI (sagittal plane) found evidence of an L4-L5 disc herniation (Date: 29.12.2022).

We present a case of a 58-year-old male patient who visited the department of neurosurgery at Acibadem City Clinic, Varna, Bulgaria, with complaints of pain initially starting from his hip, which in time extended to his left leg (L5 radiculopathy) and a few days later to his right leg (L5 radiculopathy). A physical examination was performed through the "pain leg" test, and the patient had pain in both legs upon reaching an angle above 30 degrees. Before visiting the clinic, he had been treated in Germany with physiotherapy and supplements, which had proved ineffective. An MRI discovered an L4-L5 herniated disc (Figure 1).

**Figure 2:**
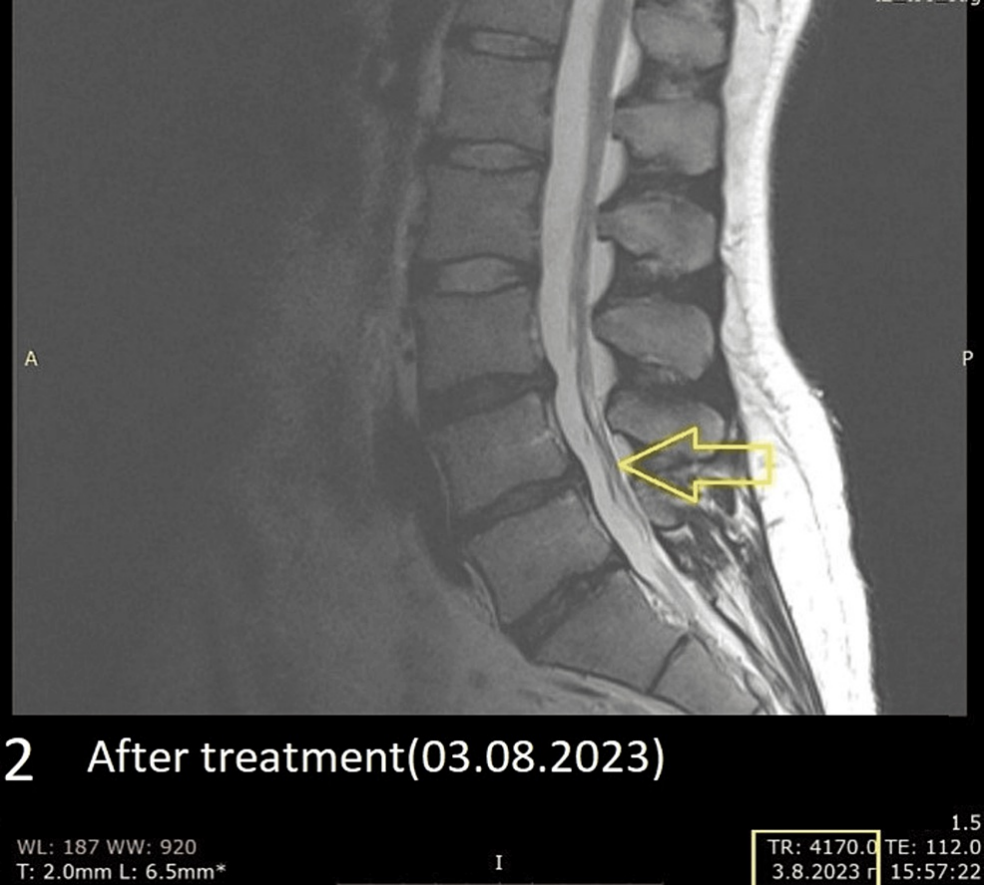
An MRI (sagittal plane) was performed seven months later without any surgical intervention; with conservative treatment, the herniation was gone (Date: 03.08.2023).

He underwent conservative treatment with nonsteroidal anti-inflammatory drugs (NSAIDs) and proton pump inhibitors (PPIs) for 14 days in addition to Medrol 4mg tablets (3x1 per day for 10 days). On the third day of the treatment, 60% of the symptoms had subsided. Upon his follow-up visit seven months later, 95% of the symptoms had disappeared. A controlled MRI revealed that the herniated disc had disappeared entirely (Figure 2).

No physiotherapy was offered because the patient had a 95% reduction in symptoms after treatment, and the follow-up MRI showed total regression of the herniated disc. The patient was able to move without pain, and therefore we decided that there was no indication for physiotherapy.

## Discussion

Lumbar disc herniations are a commonly occurring case in many pain clinics around the world [1]. Besides the elderly, more and more young people start experiencing some type of herniation by the age of 20 [1, 6]. Boden et al. conducted an MRI on 67 individuals who had never experienced low-back pain, sciatica, or neurogenic claudication. Three neuroradiologists, unaware of the subjects' clinical symptoms [2], independently interpreted the scans. Approximately one-third of the participants exhibited significant abnormalities. Among those under 60 years of age, 20% of them had a herniated nucleus pulposus, and one person had spinal stenosis. In the 60 years and older group, abnormal findings were present in about 57% of the scans [2], with 36% having a herniated nucleus pulposus and 21% showing spinal stenosis. Between the ages of 20 and 39, 35% of subjects displayed disc degeneration or bulging at least once in the lumbar region, and in the 60- to 80-year-old group, all but one individual exhibited similar issues. Given these findings in asymptomatic subjects, the conclusion drawn was that abnormalities detected in MRI should be closely correlated with age and assessed in conjunction with clinical signs and symptoms before considering operative treatment [2].

Herniated discs are divided into four main stages [7]. The first one is bulging, which is when the nucleus pulposus slightly pushes against the annulus fibrosus (outer layer of the disc) and could irritate a nerve [1, 2]. A bulging disc isn’t problematic or painful for most people, and it has the potential to resolve itself without any external or internal treatment. The second stage is called a protrusion. It is when the nucleus pulposus reaches the disc's outer layer, but on imaging, there is a more noticeable bulge, leading to a more unpleasant experience regarding pain [2]. The third stage is called disc extrusion. It is a severe development of a herniation because the nucleus pulposus goes through the annulus fibrosus and into the spinal canal, where it can move freely, therefore applying pressure to a lot of nerve structures ultimately being the cause of extending pain all the way to the lower leg [3]. At this point, patients can benefit from non-surgical treatments such as physical therapy, posture correction exercises, steroid injections, and other medicamentations and a wide range of exercises [4]. In some instances, surgery may be applicable, but in most instances, it is only needed 10% of the time. The fourth and final stage involves disc sequestration, where the inner disc material completely exits the outer shell and fully separates from the spinal disc [7]. This condition is serious and often necessitates a surgical intervention known as a discectomy. It can lead to significant complications, especially if the detached material becomes lodged in the spinal canal, causing severe nerve irritation. Consequently, patients may experience intense discomfort to the extent of being bedridden [7].

For individuals with a herniated disc unresponsive to conservative treatments, a discectomy or a less invasive microdiscectomy might be recommended [8]. Although these surgeries are generally successful, patients with a sizable opening in the outer disc ring face a substantially higher risk of reherniation post-surgery. The surgeon often determines the hole's size during the operation, and a large opening more than doubles the likelihood of requiring another procedure. A novel treatment called Barricaid (Intrinsic Therapeutics, Woburn, MA), a bone-anchored device, addresses this issue by closing the hole. In a two-year study, 95% of Barricaid patients did not undergo reoperation due to reherniation. This treatment occurs concurrently with the discectomy, within the same operation, and doesn't necessitate additional incisions or extended hospital stays [7].

Surgical intervention is required when the patient has very severe pain in their leg that hinders their quality of life, pain that has kept going for more than four weeks and is becoming stronger, and when an adequate and careful decision is made regarding the symptoms of the patient and the imaging. Operative intervention successfully reduces the pain in 90% of the patients with lumbar disc herniations. The best results are achieved two months after the first symptoms [7]. Only a small number of patients require surgical decompression [9].

Low-back pain with leg pain (sciatica) can be attributed to a herniated intervertebral disc exerting pressure on the nerve root. Lower back pain can be caused by several factors, such as age-related degenerative changes in the spine, inflammatory arthritis, disorders, or other medical conditions like accessory ossicles or muscles [10]. While conservative treatment is effective for most patients, surgical discectomy is considered in carefully selected cases to achieve faster relief of symptoms. Primary care clinicians rely on patient history and physical examination to assess the likelihood of disc herniation. Based on this evaluation, clinicians may recommend further imaging and, in some cases, surgical intervention for eligible patients. The approach underscores the importance of individualized patient assessment and treatment decisions in managing low back pain with sciatica, balancing conservative measures with surgical options for optimal outcomes [10]. Lower back pain may also be caused by osteoarthritis, degenerative disk disease, trauma, stress, and accessory structures such as ossicles, muscles, etc. [11]. In 7.5% of patients, complications can be observed. The risk of infection is 1%-5%, and that of durotomy is 5%-10% [12]. Current evidence indicates poor diagnostic performance of most physical tests used to identify lumbar disc herniation [12].

An interesting study conducted by Schvartzman et al. (1992) states that the cost of operative treatment is higher than the conservative one, but because “conservative” patients tend to be off work for a longer period of time compared to the surgically treated ones, their cost for treatment seems to be higher [13]. The most common natural course of action for a herniated disc is to heal itself. In a retrospective study by Hakelius from 1970, two groups of patients treated operatively and conservatively were followed for a period of seven years [14]. The operated group showed faster healing and faster recovery of daily activities, while the conservatively treated group showed significantly slower healing. However, their follow-up within seven years showed the same quality of life in both studied groups. The risk of leg pain recurrence was 12% in both groups [14]. In another prospective and randomized study by Weber (1983), 126 patients were followed for a period of 10 years [15]. It was found that at the end of the four-year period, there was no difference between the operated and conservatively managed groups. At the end of the first year, it was found that in 65% of the operated cases, there were satisfactory results, while in those treated conservatively, this percentage was 24% [15].

The case study, authored by Emily R. Howell, reports on the successful conservative management of a 31-year-old male recreational worker experiencing left-sided low back and leg pain attributed to a left-sided L5-S1 disc prolapse/herniation [16]. Clinical features revealed a three-to-four-month history of pain exacerbated by prolonged sitting. The intervention plan, overseen by Howell, included a comprehensive approach involving interferential current, soft tissue trigger point and myofascial therapy, lateral recumbent manual low-velocity, low-amplitude traction mobilizations, and pelvic blocking. Home care strategies encompassed heat and ice application, neural mobilizations, repeated extension exercises, stretching, core muscle strengthening, and ergonomic adjustments. The positive outcome indicated significant pain reduction after the first visit, with almost complete resolution of symptoms by the third visit. This case underscores the efficacy of conservative chiropractic care, particularly when coupled with active rehabilitative strategies, and emphasizes the importance of exploring non-surgical interventions before considering surgical referral [15].

Saal et al. conducted a study in 1990, selecting 11 patients diagnosed with lumbar disc herniation for conservative treatment and MRI follow-up. The results indicated varying degrees of protrusion absorption, with larger protrusions showing more significant reabsorption [17]. Subsequent research, including a 1996 review by Saal et al., emphasized factors influencing the natural history of lumbar disc herniation reabsorption, such as types and locations of lumbar disc herniation, anatomical factors, histochemical factors, clinical characteristics, and individual factors [18]. Meta-analyses and retrospective analyses have consistently shown a substantial incidence of symptomatic lumbar disc herniation reabsorption with non-surgical treatment [17, 18]. The North American Spine Society's (NASS) evidence-based clinical guideline also highlighted the potential for spontaneous shrinkage or degeneration of herniated intervertebral discs (IVDs) with the advancement of natural history [19]. Studies suggest that a higher proportion of nucleus pulposus may favor reabsorption, while cartilage endplate components could inhibit it [20].

The study underscores the value of customized treatment plans and the necessity of making educated decisions when managing lumbar disc herniations. By providing additional evidence of the efficacy of non-surgical treatments, it seamlessly integrates into the current literature. Consistent with earlier research on the effectiveness of conservative therapies, the study supports the notion that surgery may not be imperative in certain situations. This contribution adds to the growing body of evidence advocating for a patient-centered approach and informed decision-making in the therapy of lumbar disc herniations, reinforcing its findings with existing literature. In summary, the study makes a significant addition to the ongoing conversation about potential therapies for this common spinal ailment.

## Conclusions

This case study of a male patient, aged 58, who had a lumbar disc herniation at the L4-L5 level shows that conservative treatment led to an outstanding recovery. The research adds to the increasing amount of proof that conservative management approaches for lumbar disc herniations are effective. It emphasizes how crucial it is to create customized treatment programs that take into account both surgical and non-surgical options depending on the symptoms, imaging results, and general health of the patient. The results are consistent with earlier research, highlighting the importance of making well-informed decisions when managing this common spinal condition.
